# *AGMT3-D*: A software for 3-D landmarks-based geometric morphometric shape analysis of archaeological artifacts

**DOI:** 10.1371/journal.pone.0207890

**Published:** 2018-11-20

**Authors:** Gadi Herzlinger, Leore Grosman

**Affiliations:** 1 Institute of Archaeology, Mount Scopus, The Hebrew University of Jerusalem, Jerusalem, Israel; 2 The Jack, Joseph and Morton Mandel School for Advanced Studies in the Humanities, Mount Scopus, The Hebrew University of Jerusalem, Jerusalem, Israel; Max Planck Institute for the Science of Human History, GERMANY

## Abstract

We present here a newly developed software package named Artifact GeoMorph Toolbox 3-D (*AGMT3-D*). It is intended to provide archaeologists with a simple and easy-to-use tool for performing 3-D landmarks-based geometric morphometric shape analysis on 3-D digital models of archaeological artifacts. It requires no prior knowledge of programming or proficiency in statistics. *AGMT3-D* consists of a data-acquisition procedure for automatically positioning 3-D models in space and fitting them with grids of 3-D semi-landmarks. It also provides a number of analytical tools and procedures that allow the processing and statistical analysis of the data, including generalized Procrustes analysis, principal component analysis, a warp tool, automatic calculation of shape variabilities and statistical tests. It provides an output of quantitative, objective and reproducible results in numerical, textual and graphic formats. These can be used to answer archaeologically significant questions relating to morphologies and morphological variabilities in artifact assemblages. Following the presentation of the software and its functions, we apply it to a case study addressing the effects of different types of raw material on the morphologies and morphological variabilities present in an experimentally produced Acheulian handaxe assemblage. The results show that there are statistically significant differences between the mean shapes and shape variabilities of handaxes produced on flint and those produced on basalt. With *AGMT3-D*, users can analyze artifact assemblages and address questions that are deducible from the morphologies and morphological variabilities of material culture assemblages. These questions can relate to issues of, among others, relative chronology, cultural affinities, tool function and production technology. *AGMT3-D* is aimed at making 3-D landmarks-based geometric morphometric shape analysis more accessible to archaeologists, in the hope that this method will become a tool commonly used by archaeologists.

## Introduction

Landmarks-based geometric morphometric shape analysis is a powerful tool for the quantitative description of shape variability within and between groups. More than a decade ago, Lycett and colleagues [1] published a seminal paper that initiated a continuous rise in the application of geometric morphometric (GM) shape analysis methods to archaeological objects of material culture. Although some authors had previously attempted to apply morphometric methods, these pioneering attempts had little impact due to the limited computing power and 3-D scanning possibilities available at that time (e.g. [2, 3]). Since the work by Lycett and colleagues, several calls have been made to encourage wider adoption of these methods for the shape analysis of lithic artifacts and other objects of material culture [4,5]. These calls, which were indeed answered by an ever-growing volume of works [6–18], outlined some of the problems and difficulties entailed in the application of landmarks-based GM methods to material culture objects. The main problem was the lack of readily identifiable homologous landmarks on such artifacts, among others [5].

While solutions have been suggested for some of the problems, such as landmark homology, another substantial problem has been overlooked: the actual process of positioning landmarks and recording their coordinates [19]. In their original work, Lycett and colleagues [1] presented a protocol and an instrument, which they called a crossbeam co-ordinate caliper (CCC), for the positioning and recording of 3-D homologous semi-landmarks on lithic artifacts. While the instrument and protocol were the first to enable the application of the landmarks-based 3-D geometric morphometric (3-DGM) method to lithic artifacts, the procedure has two main disadvantages. The first is that the instrument is manually operated, and as such its operation is extremely costly in time and resources, raises serious concerns about accuracy and inter-analyst bias, and practically limits the resolution (i.e. number of landmarks) at which analysis can be conducted. The second is the physical nature of the instrument and protocol; a researcher wishing to use this method must acquire the instrument and have physical access to the studied material.

These problems had a substantial effect on the number of researchers and studies [1, 7, 14, 15, 18] applying the landmarks-based 3-DGM method. Although there are a few alternatives to the CCC for positioning and recording of landmarks, none of them provides a complete and comprehensive solution to the problems embedded in the process. One of these uses a 3-D digitizer like those produced by Microscribe [6]. However, as this too is a physical and manually operated instrument, it basically has the same disadvantages as the CCC. There are also several freely available computer programs designed for landmarks based GM analysis, such as MorphoJ or tpsDig. However, these lack 3D landmark acquisition capabilities [8–10, 13, 16]. A different computer program, which records 3-D landmarks on digital models, is IDAV Landmark [11, 17]. This software, however, was designed mainly for 3-DGM analysis in the context of biological research and as such requires that both the studied object and the landmarks be positioned manually. This renders its use inappropriate for the study of material culture objects, as it requires the use of semi-landmarks that draw their homology from an explicit, objective and reproducible geometric protocol for positioning of both objects and landmarks [1]. Lastly, the Morpho and geomorph packages for R allows the recording of 3-D semi-landmarks on digital models as well as the definition of an explicit geometric positioning protocol for both object and semi-landmarks [20]. However, these tools require proficiency in the R statistical programming language, a skill that is not commonplace among archaeologists.

**Fig 1:**
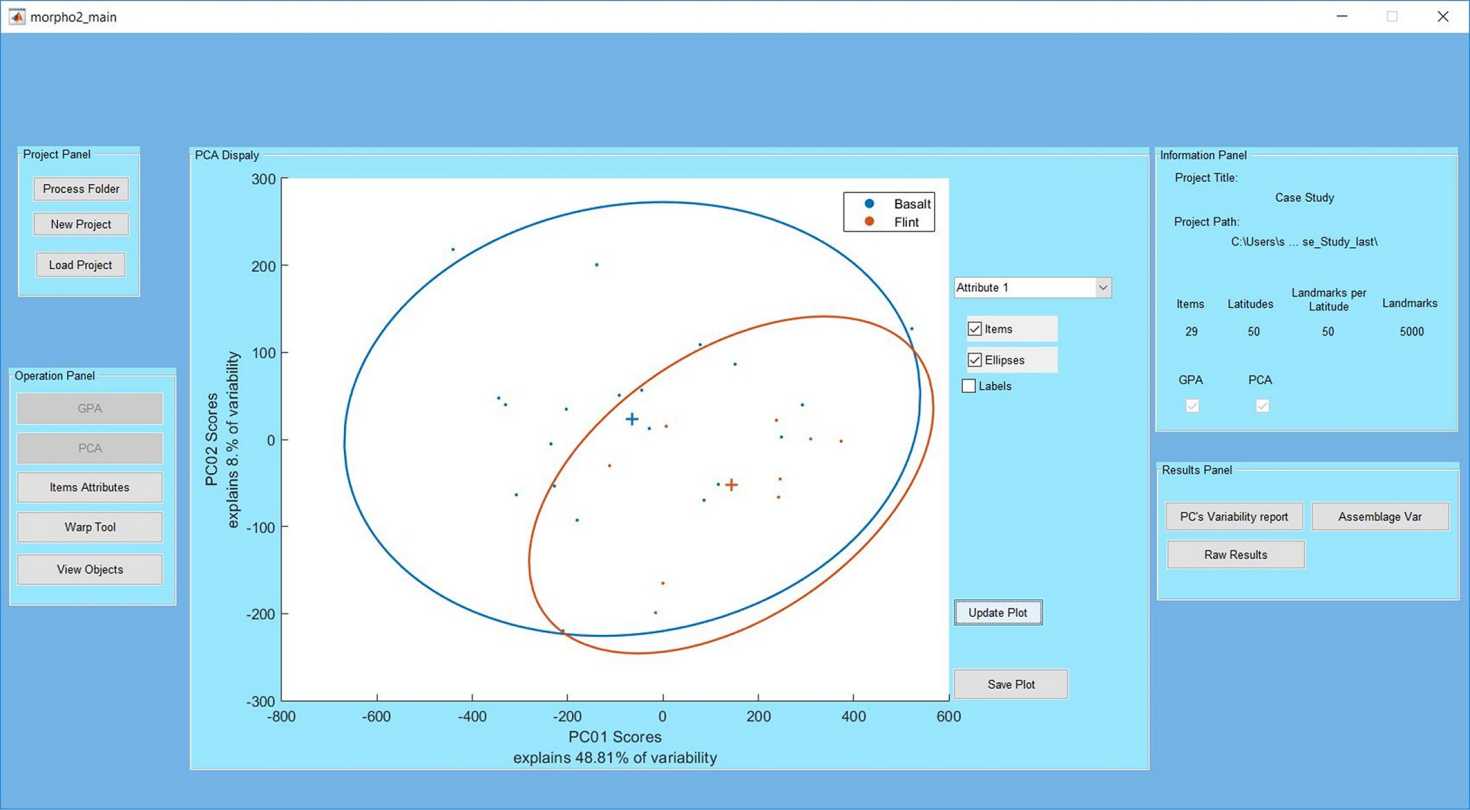
*AGMT3-D* main panel. The PCA display panel presents the scatterplot results for the case study (see below), along with color coding and confidence ellipses for the raw material groups. The information panel presents details relevant to the analysis project.

We present a newly developed and freely available computer program titled Artifact GeoMorph Toolbox 3-D (*AGMT3-D*). It was written using the Matlab programming language as a graphic user interface (Fig 1) and is provided as a standalone application for easy installation. Hence, it does not require the user to compile the code or even possess Matlab; rather, the runtime environment is automatically downloaded as part of its installation. *AGMT3-D* is designed to provide archaeologists with a simple, straightforward and easy-to-use toolbox for performing landmarks-based 3-DGM shape analysis on 3-D digital models of archaeological artifacts. The software includes an automated geometric positioning procedure for both the artifact models and the semi-landmarks, together with numerous statistical procedures for their subsequent analysis. It provides an output of quantitative, objective and reproducible results in numerical, textual and graphic formats.

## Landmarks-based geometric morphometrics: A brief overview

Landmarks-based geometric morphometrics is one variant of a group of morphometric methods used for the quantitative study of shape and shape differences between physical objects. While there are several morphometric approaches, landmarks-based geometric morphometrics is a powerful tool for the quantitative description of shape variability within and between groups [5]. This approach has been borrowed by archaeologists from various fields in biology, such as evolutionary biology and physical anthropology, where this method was originally developed and applied [21–22] (see [23] for a comprehensive review).

The method is based on a finite number of points, or landmarks, that are placed on the surface of the studied items and expressed by two or three Cartesian coordinates. These landmarks should have homology, that is, respective points should correspond across all specimens in the sample. While in biology homology can be based on phylogenetic, developmental or functional considerations, material culture objects lack such readily identifiable homologous landmarks [5, 23]. To overcome this problem, the study of archaeological artifacts usually defines semi-landmarks [24, 25, 5]. These semi-landmarks draw their homology from consistent geometric positioning of both the studied objects and the semi-landmarks. Following this approach, the present study integrates all landmark types, as well as semi-landmarks, under the general term “landmarks” [26]. To illustrate this point, one can consider two identically shaped objects. If these two objects are positioned in the exact same manner in a common space and the landmarks are positioned following the same geometric criteria, then corresponding landmarks on both items will have identical coordinates.

To describe the degree and nature of shape variability in the sample, as well as within and between sub-samples, the coordinates need to be subjected to a series of multivariate statistical procedures and analyses. The most common ones are generalized Procrustes analysis (GPA) and principal component analysis (PCA) [1, 25]. GPA serves as a superimposition procedure, removing non-shape-related variability stemming from differences in location, orientation in space and primarily scale. When this procedure is followed, differences in landmarks’ coordinates can be attributed exclusively to shape differences between different objects [25]. PCA is the main analytical procedure in the shape analysis; it is used to reduce data dimensionality and detect the main axes of variability in the sample [25]. Thus, it provides a number of components (i.e. non-correlated perpendicular axes in shape space) equal to the number of items in the sample minus one, sorted in descending order according to the proportion of variability that they explain. Each principal component (PC) reflects a specific shape trend, a mutual change in the values of a number of homologous landmarks. Each item receives a value for each PC, which is based on the values of its relevant landmarks’ coordinates in relation to the shape trend described by that particular PC. Hence, each tool is defined by a series of PC scores that describe its relative position in relation to other items in the sample for each specific shape trend. These multidimensional vectors allow the determination of the mean shape of the sample as well as those of sub-samples and their use to calculate the shape variabilities within and between sub-samples.

## Software functions

*AGMT3-D* was designed specifically for archaeologists, and as such its output is aimed at answering common archaeological research questions. Assuming that tool shapes have important implications for past human behavior, the ability to objectively and quantitatively measure and describe shape variability within and between archaeological tool assemblages is of utmost importance. Thus, *AGMT3-D* allows us to measure and describe the shape variability in an assemblage, as well as to compare the morphological nature of that variability with respect to the assemblage’s mean shape. Furthermore, it allows us to compare the means of assemblages to one another and to measure similarity and difference in the mean shapes of different assemblages. Lastly, it allows us to test the mean shape differences as well as difference in shape variabilities for statistical significance. A brief description of the main functions of *AGMT3-D* is provided below. A detailed overview and user instructions for each of the functions are available in the user manual provided with *AGMT3-D*.

### Artifact and landmark positioning

Digital 3-D models of material culture assemblages can be acquired using a variety of methods such as structured-light or laser scanning, computerized tomography and photogrammetry. Once an assemblage of models has been acquired, it can be subjected to the *AGMT3-D* for model and landmark positioning. Thus, the analysis can be executed even when the study is conducted in a different location from that of the material. The object and landmark positioning protocol of *AGMT3-D* is a modified version of a recently published protocol [27]. This protocol is almost completely automatic and requires little user involvement. First, the user is asked to select a folder containing one or more 3-D models in VRML format (*.wrl files). Next, each model in the folder is read and positioned in space in the three following steps. The first positioning step consists of moving the centroid of the object to the origin. The second step consists of its rotation about the X and Y axes to planform view, a process based on the distribution of the face normals of the model [28, 27]. The third step consists of the rotation of the object about the Z axis to maximize its bilateral symmetry, measured as the absolute difference between its outline’s negative and positive halves on the X axis. This protocol is performed consecutively for each model in the folder. It should be noted that this automatic positioning protocol best suits artifacts whose standard archaeological positioning follows their axis of bilateral symmetry, such as bifacial tools, points, arrowheads and swords.

Following this process, the user is required to review each object in a designated panel and observe and confirm its positioning. This stage is mandatory and the process cannot continue before the user confirms the positioning of each individual artifact. This is because the positioning process is based on detecting symmetries (bifacial and bilateral) and as such cannot always differentiate between archaeologically relevant aspects, such as dorsal/ventral faces and proximal/distal ends. This may cause a situation in which the surface facing “outwards” (towards the positive end of Z axis) and the end facing “upwards” (towards the positive end of Y axis) do not follow conventional archaeological positioning in all object in the sample. Therefore, the user can flip (180 degrees) objects about the Y axis and rotate (90 degrees) them about the Z axis so that all items in the sample will be positioned consistently. It should be stressed that this procedure does not modify the inherent objective positioning detected by the automatic protocol.

After the artifact positioning process is completed, the landmark positioning protocol can begin. In the *AGMT3-D* protocol the landmarks are projected onto the surface of the 3-D model in the form of a deformed grid [27]. First, the user is requested to enter the landmark sampling resolution in a grid format, that is, the number of latitudes and the number of landmarks per latitude. It should be noted that the subsequent analytical procedures require the grid configuration (i.e. number of latitudes and landmarks per latitude) of all items in the same sample to be identical. The software then deforms the grid and projects it onto the surface of the positioned model so that the latitudes are equidistantly distributed along the maximal length and the landmarks on each latitude are equidistantly placed relative to its own length, corresponding to that artifact’s width at that position. In fact, each point of the grid consists of two semi-landmarks, one placed on each of the artifact’s faces, so that a grid of 50×50 provides 5000 landmarks. The top and bottom latitudes capture the exact 3-D outline of the artifact’s distal and proximal ends. Thus, this protocol provides a list of landmarks that accurately expresses the artifact’s volumetric configuration. It should be noted that depending on the 3-D model’s resolution, the grid density and the configuration of the machine running *AGMT3-D*, this process can be somewhat time-consuming. For example, fitting a grid of 50×50 landmarks on a model consisting of about 200,000 faces using a laptop with an Intel Core i7 2.4 Ghz fifth-generation CPU and a mid-rage independent GPU takes about 15 minutes per model. However, as this part of the process is fully automatic, it does not require the active involvement or supervision of the user. Upon completion, a new file with a *.3dl extension containing the list of landmarks as well as the 3-D data of the model itself is created for each processed model. These files can then be used as input for subsequent 3-DGM shape analysis.

### Statistical processing

*AGMT3-D* can be used for statistical analysis of the landmark coordinates recorded on artifacts. Each sample subjected to analysis is managed as a separate project. By default, the results of the GM analyses are sample-specific and therefore the sample cannot be modified after it has been defined and analyzed. When initiating a new project the user is requested to provide a title and select the source data type. *AGMT3-D* supports two distinct types of source data. The first type is GeoMorph files, which can be either the *.3dl files produced by the *AGMT3-D* landmark positioning protocol or *-grid data points.mat files produced by the GeoMorph function of the *Artifact3-D* software package [19, 27]. The latter are files produced by a similar protocol that contain only landmarks’ coordinates. These two types of files can be used together in the same analysis. The second data source type is Microsoft Excel files containing lists of landmarks acquired by any of the other methods outlined in the introduction. However, for Excel landmark lists some of the graphic features of *AGMT3-D* may not function optimally, since they are not necessarily arranged in a grid configuration.

After the project has been defined, relevant information such as the project name, path, number of item, number of landmarks and grid configuration data (if available) will appear in the information panel (Fig 1). The analysis projects are automatically saved after each step in a designated folder that is created within the folder containing the data files, and can be reloaded on request. Subsequently, the user needs to perform the two main analytical procedures GPA and PCA. The results of each are automatically exported to relevant subfolders within the project folder. After completion of PCA, all the other analytical functions of the software become available. Additionally, the PC scores of all items in the sample on the first two PCs are graphically presented in a 2D scatterplot format in the main panel, along with information on the proportions of shape variability explained by each of them (Fig 1). The item’s names can be revealed by ticking the labels tick box or by clicking on any of the points.

**Fig 2:**
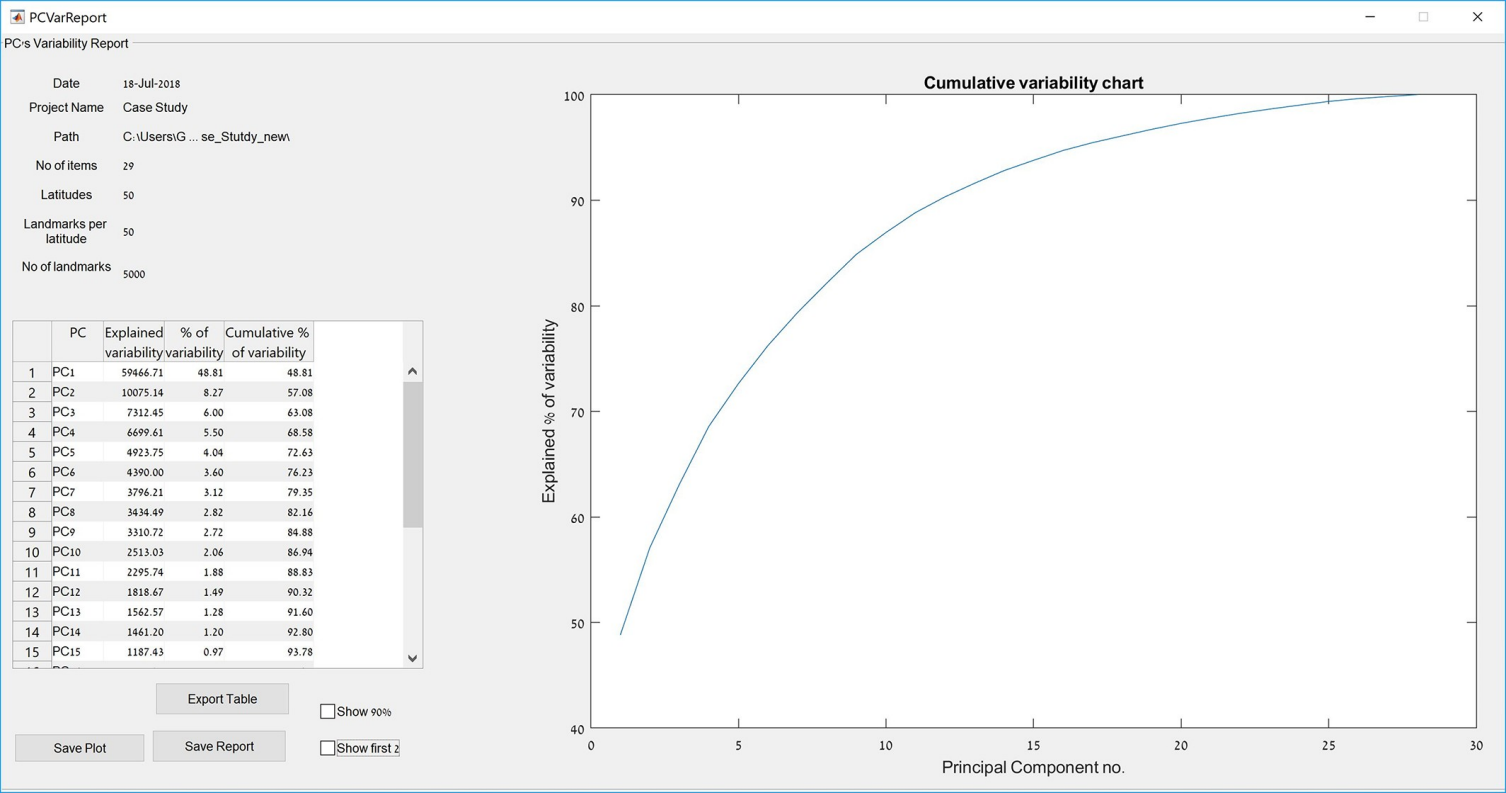
The PC variability report, showing the cumulative PC variability chart for the case study along with a table containing details on the variability explained by each of the PCs.

Additional information on the PCA results can be seen in the PC variability report panel (Fig 2). This includes the absolute and relative variability explained by each PC alongside a cumulative variability chart. The PC scores, with additional information, are automatically exported in Microsoft Excel format to the relevant subfolder in the project folder. In addition, the raw results of the analysis, consisting of the principle component score and the centroid size (prior to GPA scaling) of each artifact can be viewed and manually copied using the raw results button.

### Warp tool

**Fig 3:**
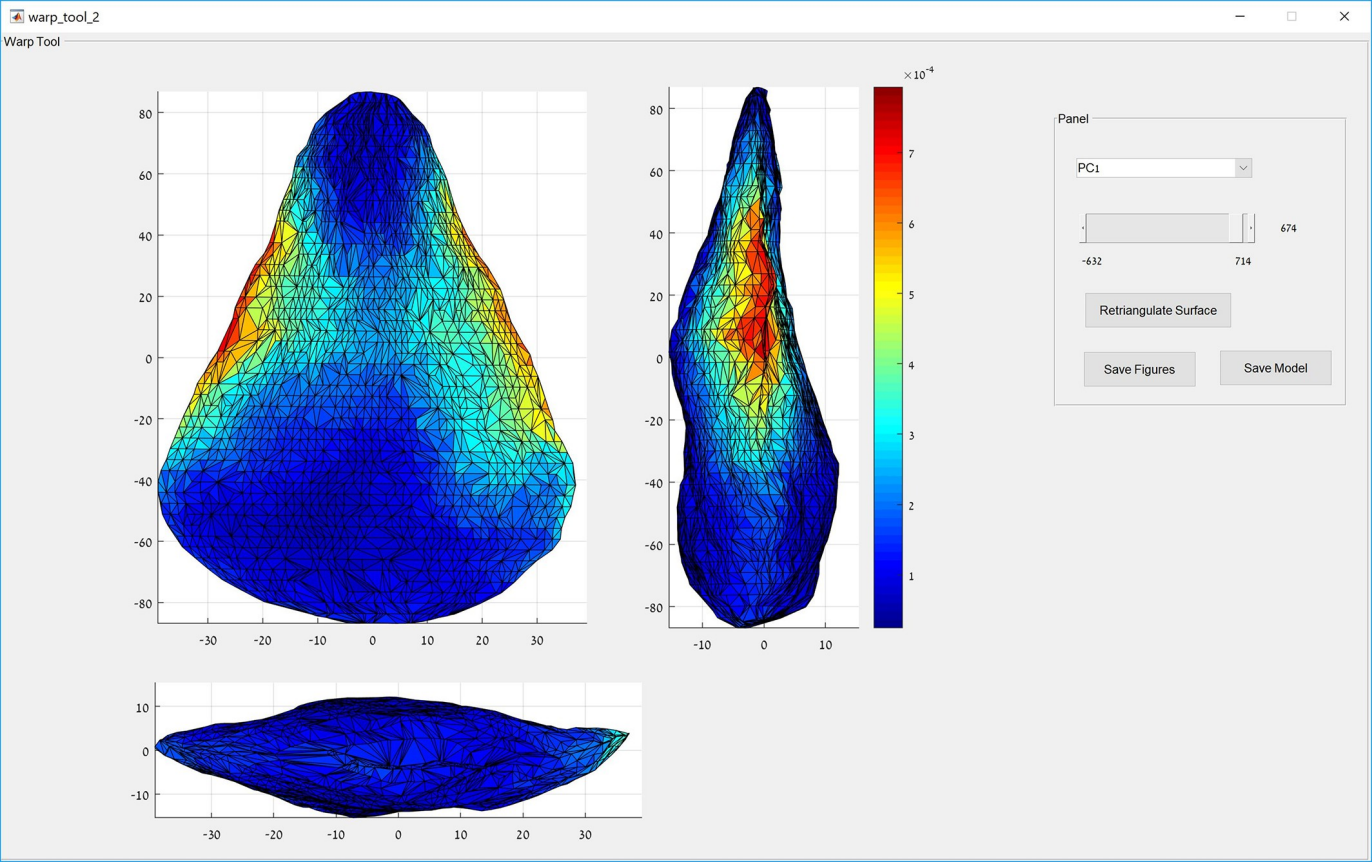
The warp tool showing the morphology of a warped hypothetical item with an extreme positive score on PC01 in the case study. Color coding represents the landmarks that vary the most on the shape trend described by PC01.

This tool allows the visualization of the shape trends described by each of the PCs (Fig 3). When this tool is opened, the user is presented with the mean shape of the sample (having a score of zero on all PCs). The mean shape is color-coded according to the selected PC to highlight the landmarks that are the most variable on that PC. The user can then modify the hypothetical score on that PC and the shape of the object will warp to express the shape of a hypothetical object having a score of zero on all PCs except for the one selected and modified. This tool can be used to visualize the shapes of hypothetical objects set at the negative and positive extremities of the various PCs, as well as to understand the nature of morphological change along the shape trends that they express. The hypothetical objects can be exported both as illustrations in high-resolution *.tif format and as 3-D models in VRML format.

### Attribute panel

**Fig 4:**
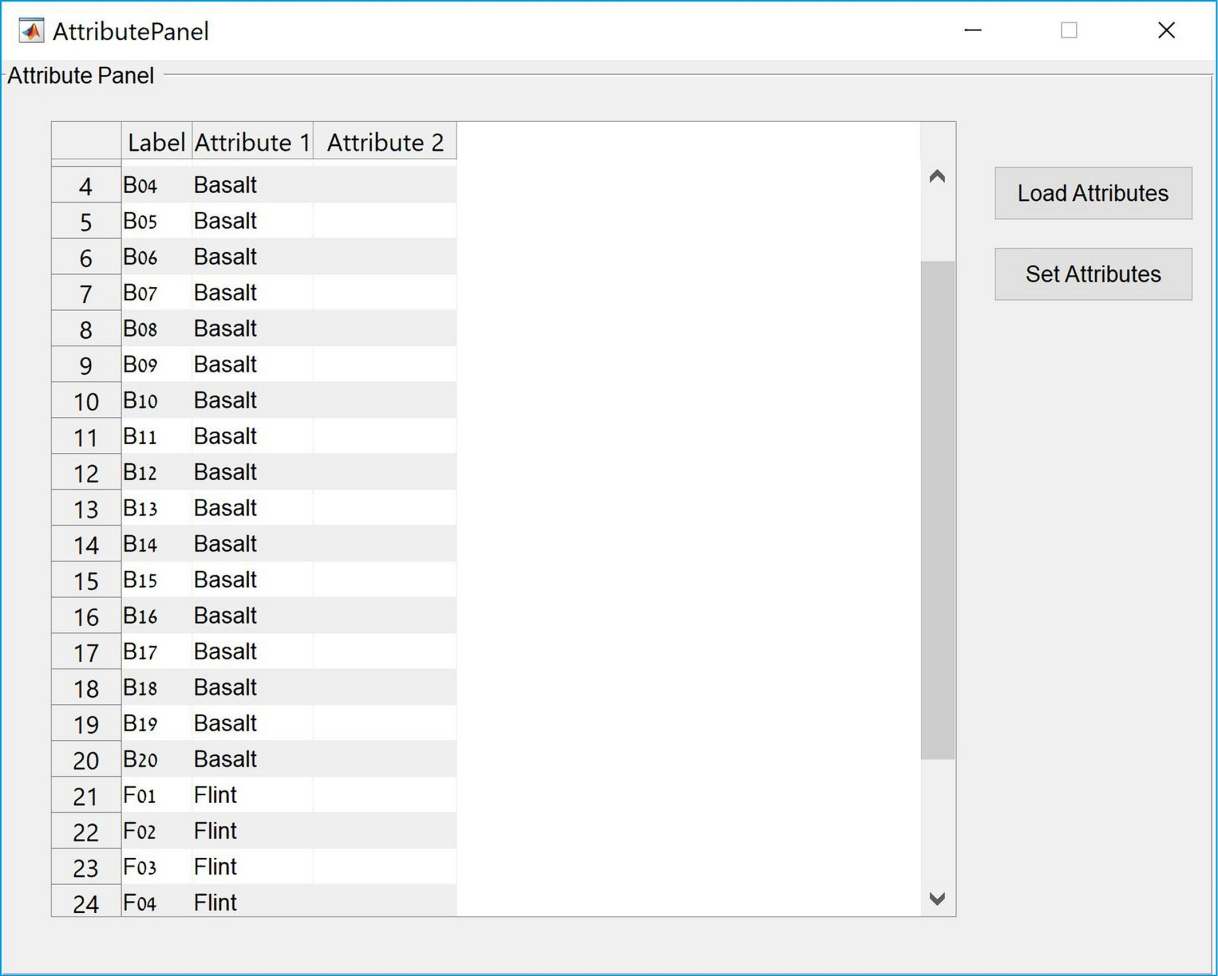
The attribute panel showing the classification of items in the case study to raw material group on Attribute 1.

This feature allows the user to assign each object in the sample to one or two predefined categorical groups in two separate attributes (Fig 4). The assignment is mandatory for the subsequent comparison of shape variabilities and mean shape differences within and between assemblages. The assignment can be performed manually or by reading the data directly from an existing Microsoft Excel file, for each of the two attributes or for both together. Following the assignment, items on the scatterplot in the main panel can be color-coded according to either of the attributes or their combinations, and 90% confidence ellipses of each group, as well as their centroids, can be plotted (Fig 1).

### Assemblage variability panel

**Fig 5:**
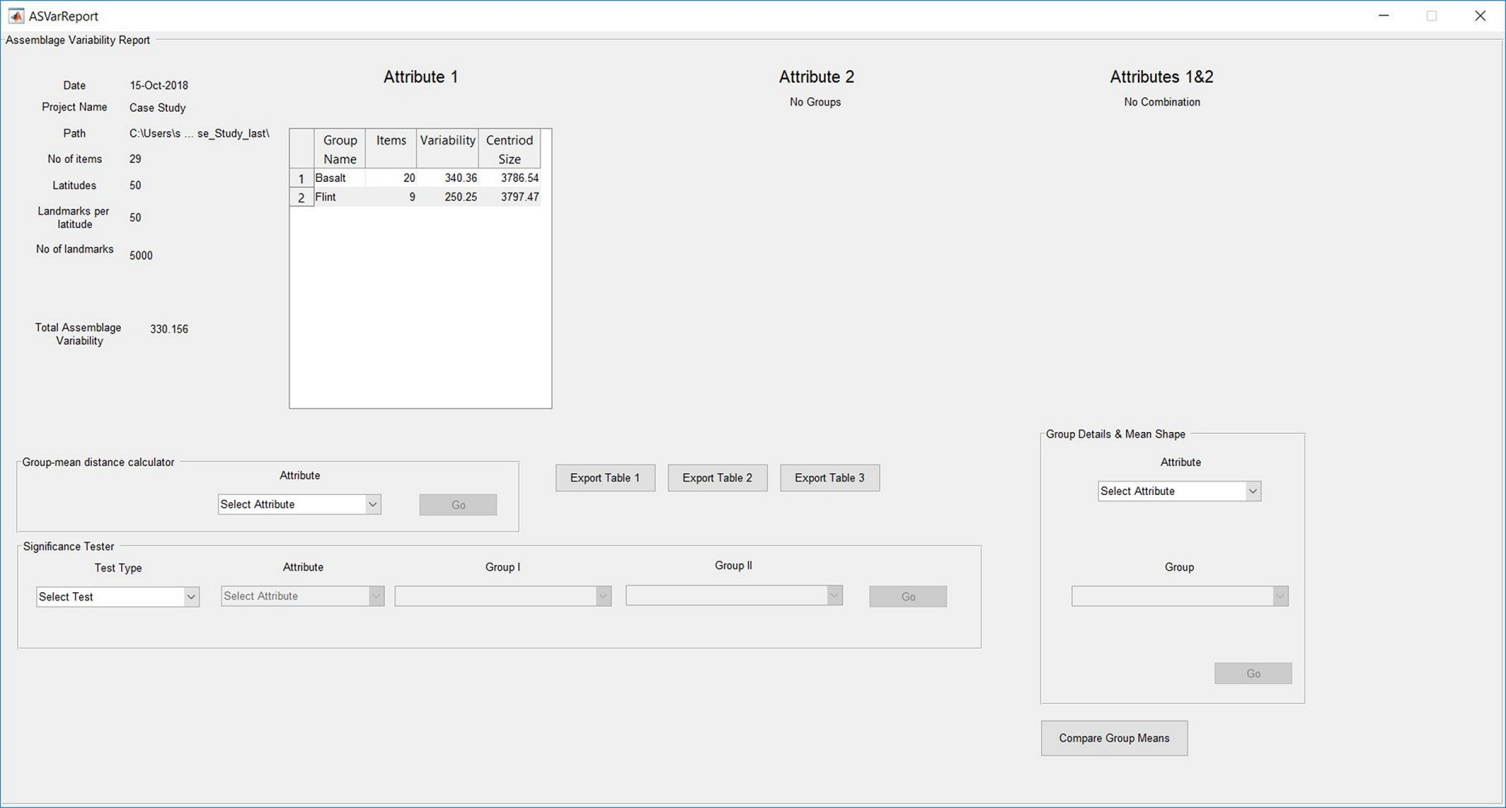
The assemblage variability panel showing the details table for Attribute1 in the case study, as well as the controls for the other analytical tools.

This panel consists of several analytical tools that test and describe variability within and between assemblages according to the predefined groups to which the items were assigned in the attribute panel (Fig 5). In the main display either one or three tables are shown, depending on the number of defined attributes. The first two tables show all defined groups in the respective attributes, while the third shows all the possible combinations of the groups. The tables show the number of items, the within-group shape variability and mean centroid size for each group. The shape variability is measured as the mean multidimensional Euclidean distance of the items in the group from the group’s centroid (i.e. mean shape). The centroid size of each artifact is measured as the square root of the sum of squared Euclidean distances of all landmarks to the item’s centroid.

**Fig 6:**
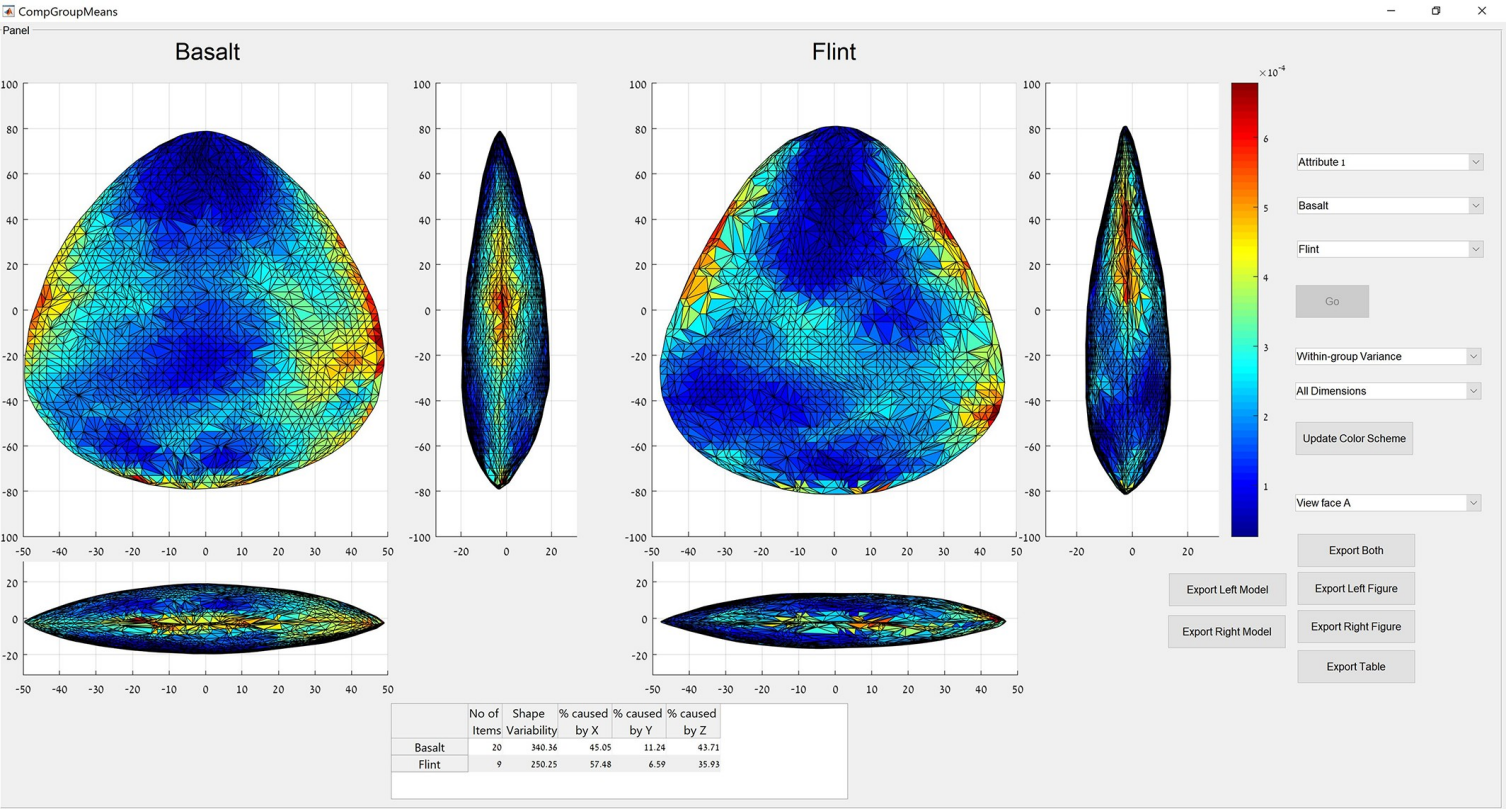
The groups’ means comparison panel showing the mean shapes of the basalt and flint groups in the case study. The color coding represents the variability of each landmark in its respective group. The table shows the percentage of variability caused by differences in each of the physical dimensions.

Another tool available in the assemblage variability panel is the “group-mean distance calculator”. This feature displays the multidimensional Euclidean distance between the groups’ centroids. The user needs to select an attribute for which a matrix of distances between the groups’ centroids and a dendrogram chart are presented. An additional feature is the “group details & mean shape tool”. This feature describes in detail the within-group shape variability. The user needs to select an attribute and one of its groups, for which a table showing the multidimensional Euclidean distance of each item from the group’s centroid as well as a dendrogram chart are presented. In addition, a visual representation of the group’s mean shape is shown, which can be exported both as an illustration and as a 3-D model. A third tool available in this panel is the “compare groups mean” tool (Fig 6). This tool allows us to compare the mean shapes of two groups graphically. The user needs to select an attribute and two of its groups for comparison. The mean shapes of these groups are then presented from three views one next to the other. The mean shapes are color-coded in accordance with the relative variability of each landmark for the items in the respective group. The user can further modify the color coding to represent the variability in only one of the three physical dimensions. Thus, users can better understand the morphological differences between the groups, not just in terms of their mean shapes but in terms of their morphological variabilities as well. The color coding can also be changed to highlight the differences in morphology between the two means. In addition, the user can choose to view the mean shapes from either of their two faces. A table that provides the proportion of variability caused by differences in each of the three physical dimensions is also shown. As with the other graphic feature, the mean shapes are exportable both as illustrations and as 3-D models.

The last tool available in the assemblage variability panel is the significance tester. This tool allows testing of the statistical significance of the equality of the shape variabilities between two groups, of the difference between the groups’ mean shapes and of the differences between their mean centroid sizes. The significance tests are performed using a Wilcoxon rank-sum test on two different sets of inter-point distances in shape space, or a set of the groups’ centroid sizes. In contrast to standard parametric significance tests such as MANOVA, this method does not require rigid assumptions regarding the distribution and variances of the populations. Furthermore, it has been shown to be compatible in cases where the number of variables are equal to or higher than the number of observations [29–30]. This method and its derivatives are increasingly applied in various research fields which encounter similar problems such as economics, astronomy and biomedical research (e.g. [31–34]).

## Case study

To demonstrate the possibilities provided by *AGMT3-D* and the new insights into archaeological investigations that it can supply, a case study is presented here. It addresses the problem of understanding the effect of raw material type on the morphological variability of Acheulian handaxes. Handaxes are among the most intensively studied stone tools in the history of archaeological research. They are the hallmark of the Lower Paleolithic Acheulian techno-complex and appear throughout the Old World for some 1.5 million years. While handaxes are perceived as a highly homogenous tool type over time and space, they also present significant variability in production technology and morphology [35–36]. The causes of this observed variability and its meaning in terms of hominin behavior have been the focus of extensive debates in the field of Paleolithic research. Among others, it has been argued that factors such as hominin cognitive development [37], cultural traditions [38–39] and knapping skills [27], as well as tool function [40], life history [41], postdepositional processes [42] and even sexual selection [43], have influenced the morphology and morphological variability of handaxe assemblages.

One of the factors most commonly claimed to affect the morphological variability of handaxes is the diversity of the raw materials used for production [44–48]. The hypothesis that the raw materials used in different assemblages have influenced their morphologies and morphological variabilities is generally based on the notion that the different physical properties of raw material types have a direct effect on the characteristics of their fracture mechanics. This may impair the knapper’s control of the result of his actions and hence also his ability to preplan and correctly execute the procedures of his reduction sequence.

This hypothesis, largely based on intuitive perceptions, is generally considered valid (but see [49] for a contradictory view). Nonetheless, to date there are no practical tools for isolating the shape variability caused by differences in raw materials. A recent work by Eren and colleagues [50] applied non-landmark-based GM shape analysis to an experimentally produced handaxe assemblage in an attempt to test this hypothesis in an objective and quantitative manner. In their work, a handaxe assemblage consisting of 105 artifacts was produced by a single expert knapper on flint, obsidian and basalt. The knapper was instructed to copy a single handaxe model in order to maintain a constant mental template. The artifacts were then subjected to a morphometric analysis that recorded 29 metrical measurements and analyzed them using multivariate statistical procedures. Their results did not detect any statistically significant differences in the mean shapes (central tendencies) of the raw material groups or in their shape variabilities. This suggests that the assumption that differences in raw material types necessarily imply differences in handaxe morphologies or morphological variabilities is unjustified.

In the current case study, we made a similar attempt to apply landmarks-based 3-DGM shape analysis to a different experimentally produced assemblage, this time using the *AGMT3-D*. The assemblage was produced as part of a larger experimental project aimed at reconstructing the *chaîne opératoire* used for biface production at the Acheulian site of Gesher Benot Ya‘aqov (GBY), Israel, which has been partially published elsewhere [51–53, 27]. Within the larger experimental assemblage, 29 artifacts were consistent with the following criteria regarding the knapper, raw material and production technology (Items B01-20 and F01-09). These are stored at the Institute of Archaeology at the Hebrew University of Jerusalem, Israel and their 3-D landmarks and mesh data are available through an online repository in *.3dl format [54]. All artifacts were produced by a single, highly skilled professional knapper, using similar technological procedures conforming to those used in the “large flake Acheulian” (LFA) technological tradition [55]. In addition, the mental template of the knapper remained relatively constant, as his final goal was to mimic the morphologically homogeneous handaxes excavated at GBY [56]. Among the 29 artifacts, 20 were produced on dense alkali-olivine basalt similar to that used at GBY and 9 on high-quality fine-grained flint collected in the Negev, Israel.

High-resolution 3-D digital models of the artifacts were acquired using a structured light 3-D scanner produced by ISRA VISION Polymetric GmbH [57]. These models were subjected to the *AGMT3-D* software’s positioning procedure for item and landmarks and were fitted with a dense grid of 50×50, resulting in 5000 recorded landmarks per artifact. Subsequently, the *.3dl files containing the landmarks’ coordinates data [54] were subjected to a 3-DGM analysis consisting of GPA and PCA using *AGMT3-D*. In total, the item and landmark positioning procedure lasted less than six hours (automatically), while the subsequent statistical analysis took less than ten minutes.

**Fig 7:**
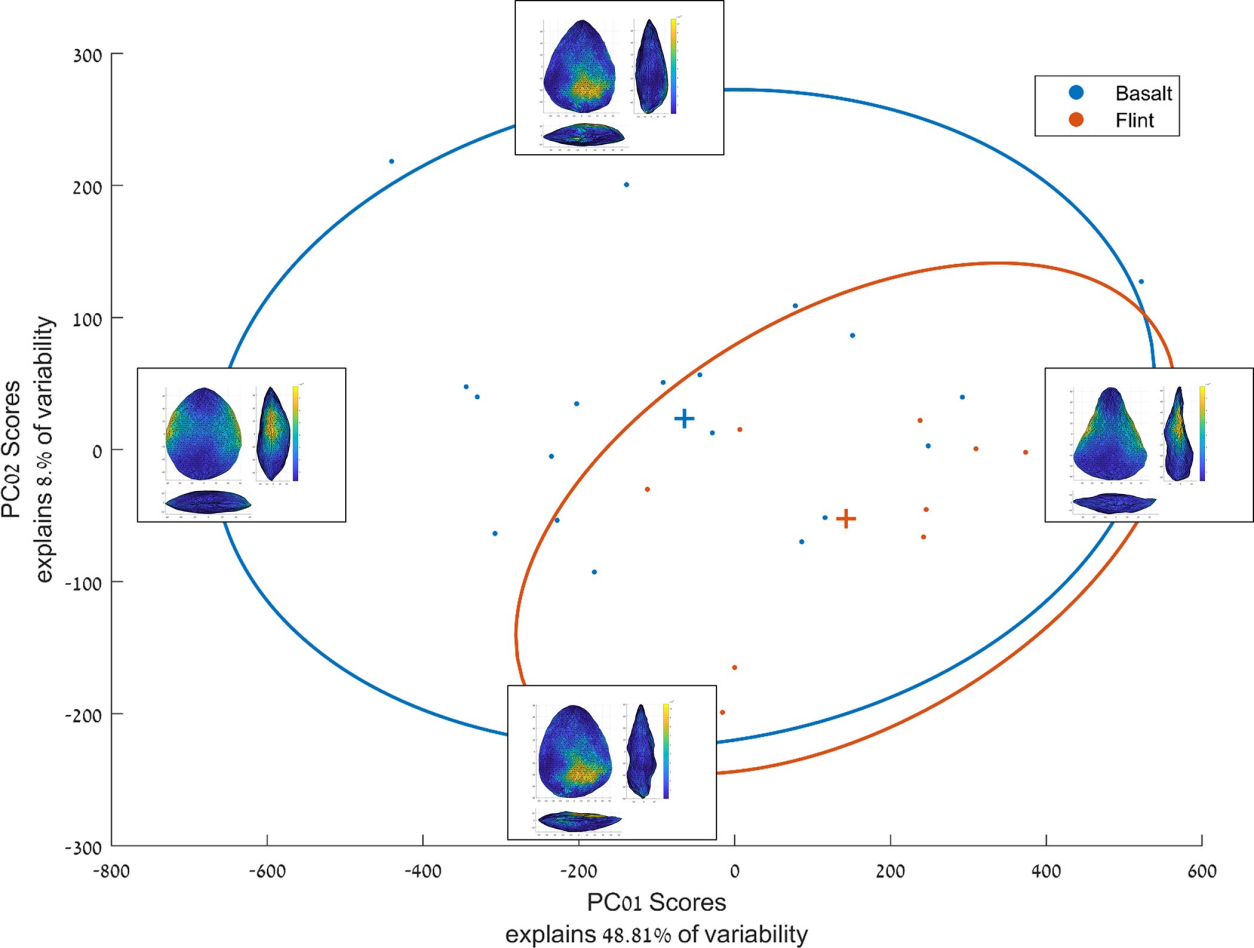
Scatterplot of the items’ scores on the first two PCs. Each point represents an item, crosses (+) represent group centroids, ellipses are 90% confidence ellipses. Artifact figures on the edges represent the shapes of hypothetical figures having a score of zero on all PCs except on PCs 1 and 2, where they have been given an extreme positive/negative score. Their color coding represents the landmarks that are the most variable on that PC.

The results indicate that the morphological variability of the basalt handaxes is some 45% higher than that of handaxes produced on flint (Table 1). This is also illustrated by the scatterplot presenting the artifacts’ score distribution across the first two PCs, explaining together about 57% of the morphological variability (Fig 7). It is evident that the flint bifaces occupy a relatively restricted area of the shape space occupied by the basalt handaxes. This area conforms to items that have straight and converging lateral edges and flat to concave ventral faces, and are more elongated. While some of the basalt bifaces also fall within this shape space, others occupy areas devoid of flint handaxes, conforming to more robust, thicker items with more convex lateral edges.

**Table 1:** Raw material groups, their within-group shape variabilities and the proportion caused by differences in each of the physical dimensions.

Group	Items	Variability	Caused by differences in X (%)	Caused by differences in Y (%)	Caused by differences in Z (%)
Basalt	20	340.36	45.05%	11.24%	43.71%
Flint	9	250.25	57.48%	6.59%	35.93%

Shape variabilities are measured as the mean multidimensional Euclidean distance between each item in the group and the group’s centroid.

While it is true that there is a substantial difference in the sample sizes of the two groups that may affect the measured shape variability, it should be emphasized that these two variables are completely uncorrelated. Adding items to a sample or removing them from it has an equal chance of increasing or decreasing shape variability. Furthermore, shape variability is measured as the mean multidimensional Euclidean distance from the centroid (in contrast to the total distance) somewhat adjusts for differences in sample size. Lastly, the observed differences can be subjected to statistical testing that takes into consideration differences in sample size. The results of a Wilcoxon rank-sum test on the inter-point distances between each of the artifacts and the centroid in each of the two groups confirm that their shape variabilities are significantly different at a .01 level (n1 = 20; n2 = 9; rank-sum: 358).

The difference in the shape space occupied by the items of the two groups is also seen in the positions of the groups’ centroids, corresponding to the mean shapes (Fig 6). When the mean shapes of the two groups are examined, they generally conform to the morphological description provided above (Fig 6). The mean shape of the flint handaxes is thinner, has a flatter cross-section, is more elongated, and has less convex lateral edges than the mean shape of the basalt handaxes. A Wilcoxon rank-sum test conducted on the inter-point distances between the mean shapes of each group and the artifacts of the opposite group indicates that the two mean shapes are significantly different at .01 level (n1 = 20; n2 = 9; rank-sum: 697). Examination of the different spatial distribution of variability in each of the groups shows that the variable areas are mostly concentrated around the lateral edges, although in the flint assemblage these areas have a more distal position. Additionally, the basalt assemblage shows greater variability in the central areas of the tool than the flint assemblage (Fig 6). Lastly, the distribution of variability across the three physical dimensions shows that in the flint assemblage the greater part of the variability stems from difference in the X dimension, corresponding to relative width (Table 1). In the basalt assemblage, relatively more variability stems from differences in the Y and Z dimensions, corresponding to relative length and thickness respectively.

In conclusion, there is significant difference between the basalt and flint handaxes in both their mean morphologies and their morphological variabilities. While some basalt handaxes show similar morphologies to those made on flint, others did not achieve the same morphology, mainly in term of elongation, straightness of the lateral edges and flatness. The shape variability measured for the basalt assemblage is 45% higher than that measured for flint.

Given that factors such as the knapper’s skill, mental template and production technology have been controlled for, these results can safely be attributed to differences in the physical properties of the raw material. The alkali-olivine basalt is much more coarse-grained than the flint and requires substantially higher energies for flake detachment. Hence, it is far more challenging to knap, allowing less control over the results of each blow. This difficulty prevented the knapper from reaching the desired morphology in some of the basalt tools, explaining the greater variability in terms of thickness and elongation.

Several factors could possibly explain the differences between the results of the current and previous studies [50]. The first is related to the production technology, since in the current study all artifacts were modified on large flake blanks produced from giant cores, while in the previous study all artifacts were directly modified on nodules. Secondly, there may be substantial variability within each of the two types of raw materials; thus differences in the qualities of the two types of basalt could have affected the knapper’s performance. Thirdly, in the current study the mental template of the knapper may have been more fluid, as no single specific copying model was used. This again could have caused the current results to be more variable (although this should have affected both raw materials equally). Lastly, the fact that the previous study applied a relatively low-resolution GM analysis (29 variables in contrast to 15,000) may have obscured some morphological variability within and between the groups. It should be emphasized that our results do not necessarily contradict those of the previous study, as its authors acknowledge that their conclusion “…does not suggest that raw material plays no role in artifact form, but instead that it cannot be assumed automatically that there are inherent lithological properties that definitively influence artifact morphology in particular ways”. In future research the sample size will be enlarged to strengthen our results.

The current example demonstrates the ability of *AGMT3-D* to apply landmarks-based 3-DGM shape analysis in a rapid and straightforward manner to provide quantitative, objective and reproducible answers to archaeologically significant questions. Thus, in light of the ever-growing accessibility to 3-D scanning equipment and computing power, *AGMT3-D* will hopefully become a widespread tool that will increase the application of this method to archaeological objects of material culture. Future developments will integrate the 3-DGM method with the manipulation of the complete 3-D morphological data to enable additional insights.

## Availability

The 3.0 version of *AGMT3-D* and its user manual are freely available for download and use under standard MIT license at https://sourceforge.net/projects/artifact-geomorph-toolbox-3d/. The software is currently available only for 64-bit machines running Microsoft Windows 7 and higher.
